# Oral cleanliness and gingival health among Special Olympics athletes in Europe and Eurasia

**DOI:** 10.4317/medoral.20396

**Published:** 2015-08-04

**Authors:** Luc Marks #, Carla Fernandez #, Imke Kaschke, Steven Perlman

**Affiliations:** 1 Dental School, Centre of Special care in dentistry, PAECOMEDIS, Ghent University, Gent, Belgium; 2 Special Olympics Germany, Healthy Athletes, Berlin, Germany; 3 Special Olympics International. Dept. of Pediatric Dentistry, Boston University, Boston(MA), USA

## Abstract

**Background:**

Special Olympics athletes, as well as the general population of people with intellectual disabilities, are expected to have poorer gingival health. The aim of the study is to explore the prevalence of gingival signs of inflammation and its relationship to oral cleanliness and age among Special Olympics athletes from Europe and Eurasia.

**Material and Methods:**

A retrospective longitudinal study was performed with data collected through standardized oral from 15.941 athletes from annual Special Olympics events held in 49 countries, from Europe and Eurasia between 2007 and 2012. The data was analysed descriptively, with One-Way ANOVA test and Chi-Square test.

**Results:**

The level of significance was predetermined at a p value < 0.05. A total of 7,754 athletes presented with gingival signs (48.64%). There were no significant differences (*p*= 0,095) in mean gingival signs between age groups, however the association between mouth cleaning and age, was statistically significant.

**Conclusions:**

The data suggests that there is a high prevalence of gingival signs among individuals with special needs; over 50% in more than 20 countries. Therefore, there is a serious need for education and preventive programs for the patients, their parents and caregivers.

**Key words:**Gingivitis, prevalence, hygiene, disability, Special Olympics.

## Introduction

Disability occurs worldwide affecting people of all ages in every country. According to the World Health Organization (WHO) 10% of the world’s population has a disability (approximately 600 million).

The concept of disability has evolved from a medical model to a social model in which environmental factors can be also considered as barriers against normal function and social integration (1). Normal function involves the ability to perform daily activities like any other individual; in this context, the topic that concerns us is the ability of a person to correctly perform personal oral hygiene (2,3).

People with disabilities are more vulnerable to oral health problems as a consequence of their oral health habits. Their oral health needs have been reported in several studies, (4-9) and the data overwhelmingly supports the fact that people with intellectual disabilities have much worse oral hygiene in comparison to the neurotypical population, (6,7) due to inability to perform adequate personal oral hygiene, causing higher levels of gingival inflammation, plaque, and periodontal disease (2,8-12).

A systematic review published in 2010 (7) studied the differences of oral health between the general population and people with intellectual disabilities. The 27 studies reported that people with intellectual disabilities have much worse oral hygiene and higher plaque levels, and a higher incidence of gingivitis and periodontitis, and overall worse oral health than the neurotypical population. (6,7)

Oral hygiene is compromised in people with intellectual disabilities due to impaired motor and cognitive skills and poor lip closure, the latter affecting the natural cleansing of the oral cavity. There are, however, other factors involved. The severity and type of disability is directly related with physical coordination, and cognitive skills of each individual, as well as the ability to comprehend and learn the importance of oral health. According to the evidence, individuals with moderate or severe intellectual disabilities have reported brushing their teeth more regularly than those with a mild disability, (13) presumably because they are dependent upon a caregiver for their oral hygiene and its frequency. The living arrangements are also considered to be a relevant factor in oral hygiene, because people living in institutions have demonstrated to have a higher prevalence of gingivitis and poorer oral hygiene (6).

Periodontal disease is an infectious disease that involves the loss of connective and bone tissue that support the teeth and gingival inflammation. The risk factors of periodontal disease include personal oral hygiene, gender, age, smoking, alcohol, diabetes and Osteoporosis, dietary calcium, stress and genetic factors (14).

The prevalence of gingivitis is reported to be 60% to 97% among individuals with intellectual disabilities compared to 28% to 75% in the neurotypical population (3). The most affected are children and adults with Down syndrome, the elderly, and those who still reside in institutions.

The direct relation between plaque accumulation and infection has been broadly studied however it is clear that the influence of other factors must also be considered. Patients with Down syndrome are known for presenting an increased prevalence of gingivitis that is related to a higher level of a specific subgingival bacterial species associated with periodontal disease (15) and impaired immunologic responses (6,10,16). As periodontal disease is marked by the permanent processes of tissue destruction and regeneration, patients with Down syndrome present with impaired gingival fibroblast motility, decreased phagocytic and chemotactic responses, altered enzymes and increased amount of PGE2; all having the potential to affect the regeneration of periodontal tissue (6,10,11,17).

Special Olympics Healthy Athletes is an initiative whose principal objective is to help athletes with intellectual disabilities to improve their health and fitness. Special Olympics Special Smiles (SOSS) is part of Healthy Athletes and it aims to collect standardized data to improve access and dental care for people with special needs (4,8,9,12,18,19).

In the absence of reliable and comprehensive international surveys of persons with intellectual disabilities, the SOSS program provides a unique opportunity to conduct a large number of standardized examinations. The indices reported include basic, epidemiological and clinical data allowing countries to identify the oral health needs of this population (20).

The purpose of this study is to explore the prevalence of gingival signs of inflammation and its relationship to oral cleanliness and age among people with intellectual disabilities from Europe and Eurasia. The data, obtained from 49 different countries, will contribute evidence to develop oral health policies and interventions in relation with oral hygiene and gingival health.

## Material and Methods

A retrospective longitudinal study was performed with data collected through interviews and oral examinations from athletes participating in the annual Special Olympics Special Smiles events held in different European countries between 2007 and 2012. The organization includes national, regional and local events as well as World Games, thereby the national data from the 51 countries was collected in different years. The athletes were examined with informed consent obtained from the athlete and a parent or guardian. The Joint Ethical Committee of the Ghent University Hospital approved the study.

Athletes with intellectual disabilities from 51 countries throughout Europe and Eurasia From these data only countries where at least 20 athletes were screened were selected. Therefore, a dentists performed the data collection for which they were previously trained and calibrated according to the Training Manual for Standardized Oral Health Screening created by the US Centers for Disease Control and Prevention (CDC) (5).

The process included athlete registration, the oral screening, and oral hygiene education (4). The strict CDC protocol keeps data collection in a simple multi center setup where each athlete is examined by a trained and strict calibrated dentist.

The frequency of oral hygiene was determined by the question, “How often do you clean your mouth?” The possible answers were; once or more a day, 2 to 6 times per week, once per week, less than once per week, and not sure (9,12,21).

The presence of gingival signs was defined as “free or attached gingival margins or papillae that are moderately red, or showing significant deviations from normal contour or texture, occurring on three or more permanent teeth within the buccal area of the mandibular arch, cuspid to cuspid”(5,9,18,22).

The data collected was compiled and transferred to an SPSS data file and descriptive parameters were obtained. The data from countries with larger sample sizes (Belgium, Germany, Italy, Poland, and Romania) was divided in three age groups, namely, “under 18 years,” “between 18 and 25,” and “26 and over.” This new data was analyzed with One-Way ANOVA test and Chi-Square test was performed to assess the relationship between age group and frequency of mouth cleaning. The level of significance was predetermined for all the tests and performed at a *p*<0.05.

## Results

A total of 15,941 subjects from 49 countries were considered for the present study. The amount of athletes screened per country had a minimum of 20 (Montenegro and Georgia), median of 54 (Isle of Man and Austria), and maximum of 3,584 (Germany). The mean age of the subjects was 28.5 years with a std. deviation of 5.9 years and there were 6,012 females (37.7%) and 9,878 males (61.97%).

**Figure 1:**
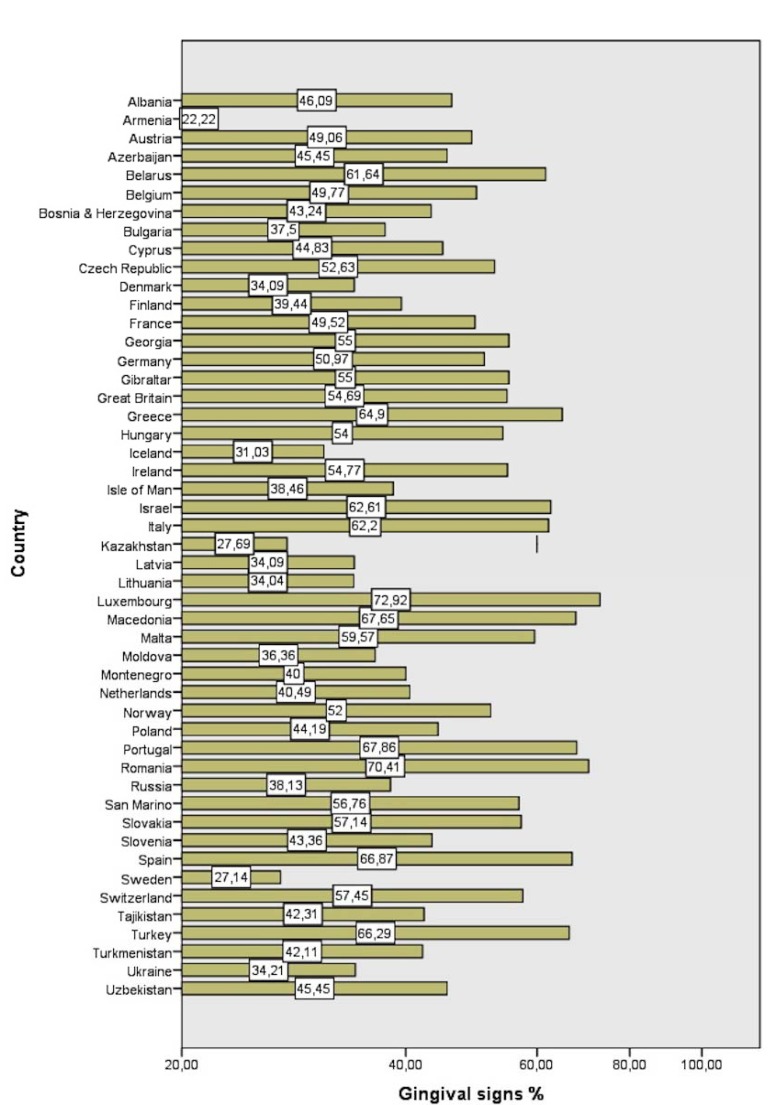
Prevalence of gingival signs per country (%).

A total of 7,754 athletes presented gingival signs of disease (48.64%) with a std. deviation of 12.48%. The highest prevalence was found in Luxembourg (72.92%), Romania (70.41%) and Portugal (67.86%). The three countries with the lowest prevalence of gingival signs were Armenia (22.22%), Sweden (27.14%) and Kazakhstan (27.69%) (Fig. 1).

**Figure 2:**
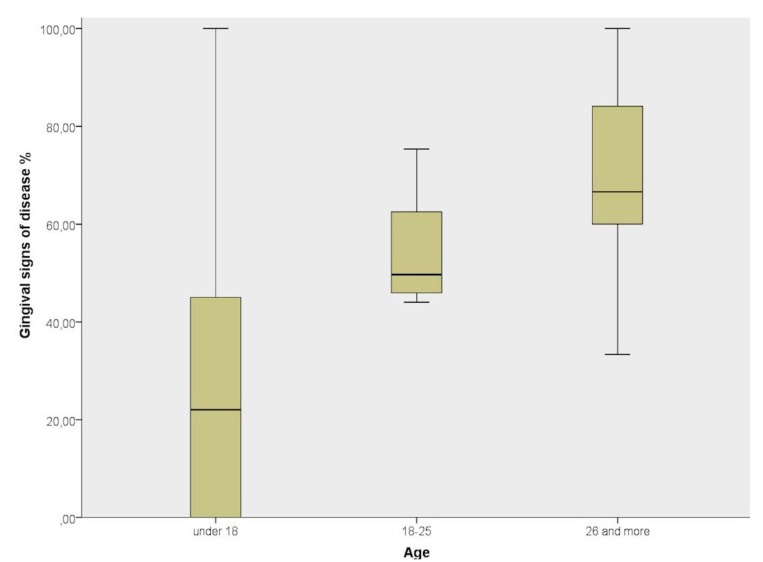
Distribution of gingival signs prevalence per age group.

When comparing the different age groups (under 18, between 18-25 and over 26), the One-Way ANOVA (F=2,768, *P*=0,095) and Multiple Comparisons LSD tests showed that there were no significant differences in the mean gingival signs between the three age groups (Fig. 2).

**Figure 3:**
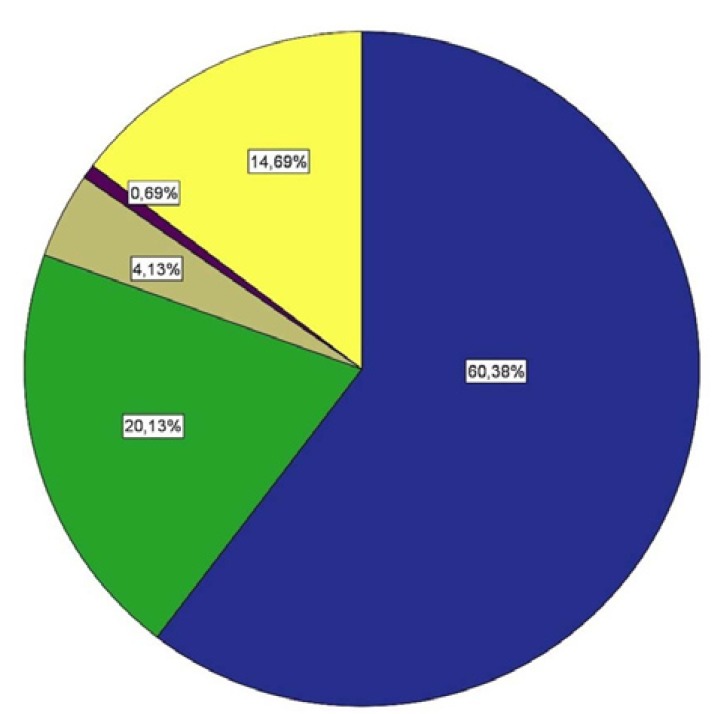
Overall frequency of mouth cleaning. Once or more a day (blue), two to six times per week. (green), once per week. (ivory), less than once per week.(purple), not sure (yellow).

Figure 3 shows a pie chart with the oral hygiene behaviour, (Blue: Once or more per day; Green: Two to six times per week; Brown: Once per week; Purple: Less than once per week; Yellow: Uncertain.) 60.38% of athletes cleaned their mouth at least once per day and 20.13% two to six times per week.

A 56.7% of the group of age “under 18” and 98.31% of the group “between 18-25” reported cleaning their mouth more than once a day. In the group of “26 an over”, however, 46.27% cleaned their mouth more than once a day and 44.71% two to six times a week. With values of Pearson Chi-square 1555, *p* = <0.001 and Phi= 0.986, the association between mouth cleaning and age was statistically significant; the older athletes brushing their teeth less frequently.

## Discussion

The standardized SOSS screening protocol has been widely reported in the literature since 1995, (4,9,18,21-23) but analysis of this data must be made with caution. The participants in the study were relatively young with a mean age of 28.5 years, therefore this sample cannot be considered representative of all people with intellectual disabilities because they consisted of a younger, healthier, higher-functioning and better supported stratum of that population (5,24,25) The relevance of this, is that the oral cleanliness and presence of gingival signs in the rest of this population would be expected to be worse. It is also possible that some athletes could have participated in more than one event during the years of the data collection. Thereby, bias in data collection may exist but there are no means in the study to determine it (19).

This study, nevertheless, has several strengths. It provides evidence from a large unique sample size, from multiple geographic regions and obtained by standardized procedures. The CDC protocol was developed to ensure standardized data collection. The standardization training consists of an oral or online presentation with exercises and a test question-answer period. Moreover, questions are asked in a simple yes/no format. The set up of the protocol reduces bias due to screener variability and allows the results to be consistent, valid, and reliable. Intra- and inter-observer concordances, however, are not included in the protocol. The main advantage of this procedure is that standardization allows data from several sites to be combined with past, present and future data from Special Olympics Screenings worldwide. In this case the data of almost 16,000 athletes from different countries is reported, and can be used as a baseline in determining the needs of people with intellectual disabilities in relation to oral cleanliness and gingival inflammation.

From this data, gingival signs were reported in 48.64% of the 15,941 athletes with an intellectual disability from 49 countries throughout Europe and Eurasia. The prevalence varied widely between countries (22.22%-92.72%). The latter could be explained by differences in sample sizes. The five countries with larger sample sizes such as Poland, Germany, Romania, Italy and Belgium showed a prevalence of more than 50%, which is remarkable considering that this study was based only on the examination of the gingiva within the buccal area of the mandibular arch, cuspid to cuspid, and the permanent dentition according to the CDC protocol. Moreover, the result was positive when at least three or more teeth presented gingival signs. This prevalence of gingival signs contrasts strongly with the reported frequency of mouth cleaning where 60% of the athletes declared brushing their teeth once or more a day. This apparent contradiction between self-reported and screened data can be related with the athlete’s ability to perform adequate personal oral hygiene and their comprehension of the self reported questions.

The results obtained are comparable with other studies based on data from special Olympics in United States 2001 (40.1%) (12) (Formatting Citation)(Formatting Citation), Puerto Rico (42%) (21), Venezuela (45%) 2013 (21), UK 2005 (63%) (19), Italy 2009 (60 %) (22) and Mexico 2013 (21) (52%).

Existing evidence indicates that the prevalence of periodontal disease is lower in young individuals than in adults; however in this study age was not significantly related to the prevalence of gingival signs even though the prevalence increased with age. Age did show a strong relation with mouth cleaning behavior, although this data was self-reported and could have been influenced by the previous knowledge of the ideal frequency of mouth cleaning. Most of the younger athletes reported cleaning their mouths once or more times a day, which is very positive even though the effectiveness of technique was not measured. It is also relevant that even when this data relates to higher-functioning individuals with intellectual disabilities, almost the half of the older athletes did clean their mouths every day. Nevertheless, far worse values would be expected from lower-functioning athletes and this evidence demonstrates the need for educational programs for prevention.

The severity of disability has an obvious influence over cognitive and motor skills and may limit the ability to comprehend or perform personal oral hygiene making it necessary to rely on a caregiver for supervision or assistance. Caregivers who perform daily oral hygiene should be trained in order to perform this task, (2) because frequency of cleaning is not directly related to effective plaque removal and oral health preservation (26).

The devices used to perform oral hygiene at home were also not considered in this study. Some published studies have demonstrated the advantages of power assisted toothbrushes for removing dental plaque in people with intellectual disabilities and have proven to be significantly helpful (27,28).

Another relevant aspect is the frequency of professional dental care. Those individuals who periodically receive dental care should be expected to have better oral hygiene and less gingival signs. When people with disabilities and/or their caregivers seek dental care, access is affected by many factors. It is beyond the scope of this article to review all these aspects, however they should be considered in order to understand the different barriers that confront this vulnerable population that together with all other factors considered are responsible for the need for improved oral health.

People with intellectual disabilities are reported to have a higher prevalence of gingival signs, principally being affected by age, behaviour and, type and severity of disability. In this study, the mean prevalence of gingival signs of disease was 48.64%, but over 50% in more than 20 countries. Therefore, it appears that a high percentage of this population is in need of education and preventive programs for the patients, their parents and caregivers. Improvements in these indicators will have a strong impact in the oral health and quality for life of this people with intellectual disabilities.
